# ActiveReach: an active learning framework for approximate reachability query answering in large-scale graphs

**DOI:** 10.3389/fdata.2024.1427104

**Published:** 2024-11-19

**Authors:** Zohreh Raghebi, Farnoush Banaei-Kashani

**Affiliations:** Department of Computer Science and Engineering, University of Colorado Denver, Denver, CO, United States

**Keywords:** reachability query, reachability learning, index learning, graph learning, graph mining

## Abstract

With graph reachability query, one can answer whether there exists a path between two query vertices in a given graph. The existing reachability query processing solutions use traditional reachability index structures and can only compute exact answers, which may take a long time to resolve in large graphs. In contrast, with an approximate reachability query, one can offer a compromise by enabling users to strike a trade-off between query time and the accuracy of the query result. In this study, we propose a framework, dubbed ActiveReach, for learning index structures to answer approximate reachability query. ActiveReach is a two-phase framework that focuses on embedding nodes in a reachability space. In the first phase, we leverage node attributes and positional information to create reachability-aware embeddings for each node. These embeddings are then used as nodes' attributes in the second phase. In the second phase, we incorporate the new attributes and include reachability information as labels in the training data to generate embeddings in a reachability space. In addition, computing reachability for all training data may not be practical. Therefore, selecting a subset of data to compute reachability effectively and enhance reachability prediction performance is challenging. ActiveReach addresses this challenge by employing an active learning approach in the second phase to selectively compute reachability for a subset of node pairs, thus learning the approximate reachability for the entire graph. Our extensive experimental study with various real attributed large-scale graphs demonstrates the effectiveness of each component of our framework.

## 1 Introduction

Graphs have evolved into a general conceptual abstraction that can model complex connections between objects. Developing a scalable method for the analysis of large-scale graphs, such as biological, road, and social networks, is a challenging task. An interesting graph analysis application is to verify whether a vertex is reachable from another. Reachability queries have been used extensively in diverse applications, including social networks, biological networks, and communication networks.

Given a graph *G* with vertices *V* and edges *E*, one extreme solution to answer reachability queries is to pre-compute the full transitive closure of *G*. The transitive closure (TC) of a graph *G* = (*V, E*) is a graph *TC* = (*V, E*+) such that for all *v*, *w* in *V* there is an edge (*v, w*) in *E*+ if and only if there is a path from *v* to *w* in *G*. By pre-computing the TC, one can answer reachability queries very efficiently. However, TC requires a large space and computing TC is very time-consuming; hence, using TC is a very expensive solution for large graphs (Yildirim et al., 2010). On the other extreme, one can use depth first search (DFS) or breadth first search (BFS) of the graph. With DFS or BFS, we traverse the input graph from query source node until the destination node is reached, or it is found that no path from source to destination exists. This approach requires no pre-computation but takes *O*(*V*+*E*) time to answer a query, which is impractical for large graphs. Existing index-based solutions (Yildirim et al., 2010; Agrawal et al., 1989; Wang et al., 2006; Chen, 2009; Jagadish, 1990; Veloso et al., 2014; Zhou et al., 2017; Seufert et al., 2013; Bramandia et al., 2008; Jin et al., 2009; van Schaik and de Moor, 2011; Jin et al., 2012; Cheng et al., 2015; Schenkel et al., 2005; Tri-ssl and Leser, 2007) trade off pre-computation and online search, compromising index construction time and space for query time. The main purpose of these methods is to sufficiently pre-compute reachability information to guide online search for query processing. However, exact reachability query answering using such index-based solution is still too time-consuming with high memory usage to be practical in large-scale graphs for many applications. For such applications, a quick answer that approximates the query result may be preferred as a practical compromise. This is particularly useful solution for applications that do not require exact answers in the first place. Unfortunately, existing index-based reachability query processing methods spend several minutes or hours to provide answers, even for moderately sized graphs (Iyer et al., 2018b; Zhang et al., 2007; Iyer et al., 2018a).

### 1.1 Applications

Surprisingly, approximate reachability arises in a variety of scenarios. In network security, it is just sufficient to have a rough estimate of the probability of reachability to specific file systems (Muñoz González et al., 2017). Another application known as influence maximization, whose main application is viral marketing (Zhu et al., 2017; Jin et al., 2011), with approximate reachability we can determine nodes that can be influenced by a given set of nodes without spending lots of resources to calculate exact answer (Kempe et al., 2003; Zhao et al., 2011; Iyer et al., 2018b). In the study of viral disease epidemics, transmission of the virus from a group of carrier individuals to a group of receivers can be answered with approximate reachability (Raghebi and Banaei-Kashani, 2018). Instead of pre-computing the entire reachability set, we can consume less storage and computation resources to approximately answer the reachability queries. In mobile networks and routing applications, we can determine the probability of receiving a packet from the source node to the destination node (Ghosh et al., 2007).

Toward this end, in this study we introduce *ActiveReach*, a learning-based framework to learn reachability for processing approximate reachability queries in large graphs. To the best of our knowledge, we are the first to introduce approximate reachability queries and propose a learned index for efficiency answering of such queries. Our proposed learned reachability indexes are subject to the same advantages and disadvantages as previously proposed learned index structure (Kraska et al., 2018). However, we argue that learning index structures that can accurately predict reachability are particularly a suitable approach for processing approximate reachability queries.

With ActiveReach, we calculate embeddings for nodes to preserve reachability information. ActiveReach utilizes three key types of information to learn reachability. First, ActiveReach leverages the concept that nodes sharing similar attributes are more likely to be reachable. For example, in social networks, if two members exhibit similar interests and attributes, the probability of them belonging to the same communities and being reachable is significantly higher (Backstrom et al., 2006). Second, ActiveReach considers that nodes that are closer in terms of graph distance are more likely to be reachable (Kempe et al., 2003). Finally, incorporating reachability information from the graph can enhance the reachability learning process. ActiveReach is designed based on these fundamental principles.

ActiveReach is a two-phase framework that computes embeddings for attributed graphs using position-aware graph neural networks in the first phase. In the second phase, ActiveReach utilizes pre-computed embeddings as node attributes and employs an active learning solution. This solution actively selects representative pairs of nodes to calculate reachability between them to be added to the training set to improve reachability prediction performance. The more effective the method is in selectively computing reachability given a fixed budget, the better the learned model will perform. We consider three strategies for selecting pairs of nodes to be labeled. These strategies include reachability prediction uncertainty, graph structure information, and information density in the embedded space.

To summarize, we make the following contributions in this study:

- We define approximate reachability query.
- We introduce a framework to learn new reachability index structures for approximate reachability query processing efficiently.
- We introduce an active learning strategy to select informative and representative nodes to calculate reachability and label data.
- We perform an extensive experimental study with various sets of graph datasets to evaluate the performance of our proposed solutions for approximate reachability query processing.

The remainder of this study is organized as follows. In Section 2, we review the related work on reachability query processing, graph embedding, active learning, and index structure learning, in relation to our proposed algorithm. In Sections 3, 4, we formalize our problem, and in Section 5, we present our ActiveReach algorithm. In Section 6, we present our experimental results, and finally, we conclude the study and discuss future directions of this study in Section 7.

## 2 Related work

### 2.1 Reachability query processing

In this section, we present the body of literature on reachability query processing on graphs. At one extreme, the transitive closure (TC) of the input graph can be precomputed to answer reachability queries. With this approach, since reachability between all pairs of vertices is precomputed, reachability can be answered in *O*(1). However, this requires *O*(*n*^2^) storage for large-scale graphs, where *n* is the size of the graph vertex set. On the other hand, DFS (Depth First Search) and BFS (Breadth First Search) can be used for reachability query processing without pre-computation. These approaches do not leverage pre-computation and fail to scale for large graphs. To balance query time and size of the pre-computed reachability, various families of index-based solutions are proposed in the literature that maintains a compact version of the transitive closure (Agrawal et al., 1989; Jagadish, 1990; Jin et al., 2008; Wang et al., 2006; Jin et al., 2012; Cohen et al., 2002; Wei et al., 2014; Bramandia et al., 2008; Jin et al., 2009; Sengupta et al., 2019; Jin and Wang, 2013) and/or improve efficiency of online search for reachability query processing (Yildirim et al., 2010; Jin et al., 2008; Seufert et al., 2013; Su et al., 2017; Wei et al., 2014). Some of these methods are efficient while they use large memory to be fast, and some of them are slow while memory-wise efficient. However, in our algorithm, we use a user-defined parameter to select a portion of the data as the training set and we apply graph neural networks to learn reachability. We briefly review these families of index-based solutions for reachability query processing below.

#### 2.1.1 Interval labeling

A large family of reachability query processing methods relies on interval labeling, where a complete interval set (related to tree and non-tree edges) is assigned to each vertex to encode the set of vertices reachable from each vertex (Agrawal et al., 1989; Wang et al., 2006; Jin et al., 2012; van Schaik and de Moor, 2011; Tri-ssl and Leser, 2007; Shirani-Mehr et al., 2012; Raghebi and Banaei-Kashani, 2018). Comparative studies (Veloso et al., 2014; Seufert et al., 2013) have shown that while fast in query answering, the large size of the index structures required for interval labeling is a major bottleneck in scalability of these methods for large graphs.

#### 2.1.2 HOP labeling

Another family of reachability methods is based on HOP labeling (Bramandia et al., 2008). With this approach, TC is encoded recursively by maintaining the reachability information in a distributed manner distributed across graph nodes (Bramandia et al., 2008; Jin et al., 2009; Schenkel et al., 2005; Su et al., 2017; Jin and Wang, 2013; Wei et al., 2014). With these methods, each vertex records lists of vertices reachable from the vertex (out-reach) as well as a list of vertices reachable to the vertex (in-reach). The main drawback of this family of methods is the long index construction time. Sengupta et al. (2019) and Leskovec et al. (2005) use random walk to answer reachability query. In Sengupta et al. (2019), they generate in-reach and out-reach sets for source and destination query nodes using random walks during query processing. They do not precompute any index structure and during query processing apply first-order random walks to answer reachability queries. They consider that the next node to be visited is only impacted by the current node. But in many real-world applications such as web-based graphs, next page visit is not only based on the previous visit but also impacted by the sequence of last clicks. This type of relationship is called higher-order dependencies. The first-order random walk does not capture higher-order dependencies (Tang et al., 2015; Ou et al., 2016). In addition, to have an accurate result, this method should generate long enough random walks so that with a reasonable number of random walks, two reachable nodes are visited. This category of methods is very time-consuming to run especially for sparse graphs.

#### 2.1.3 Fast online search

This family of methods (Yildirim et al., 2010; Seufert et al., 2013) focuses on speeding up the online search rather than pre-computing reachability. Toward this end, the online search methods create partial labeling information for each vertex and utilize this information to reduce the query time by pruning the search space. Unlike interval labeling and hop labeling which pre-compute reachability information comprehensively, in fast online search only partial reachability information is stored during index construction to be used during query processing. A prominent representative in this family is GRAIL (Yildirim et al., 2010). GRAIL assigns each vertex multiple reduced interval labels where each interval is generated with random DFS traversal of a graph. The interval is used to determine whether a vertex can be pruned when it cannot reach the query target vertex. Wei et al. (2014) and Su et al. (2017) are another category of research which use k-min-wise independent permutation and bloom filter labeling, respectively, to encode in-reach and out-reach set in a way to prune the search space similar to GRAIL during query processing.

#### 2.1.4 Reachability backbone

Methods in this family introduced the concept of reachability backbone to improve the scalability of the traditional reachability indexes including interval labeling and hop labeling approaches (Jin et al., 2012). In this study, the main idea is to identify and pre-compute reachability information for a subset of the graph (i.e., the backbone) that can most inform reachability queries for the entire graph. SCARAB (Jin et al., 2012) is one of the representatives of this family. To answer reachability query between two vertices, SCARAB verifies whether and how query vertices have access to the backbone vertices and then searches the backbone to resolve reachability between access points to the backbone. Since the size of the reduced backbone graph is small, one can use a different index-based structure to process backbone reachability queries. For instance, SCARAB uses GRAIL (Yildirim et al., 2010) for reachability query processing on backbone.

In Table 1, we show comparison summary between traditional index-based solutions. In this table, “High” refers to the analysis complexity of *O*(*n*^2^) and above, and “Low” refers to constant to linear time complexity. For ActiveReach, complexity depends on the query budget and graph embedding is computed in an offline manner.

**Table 1:** Comparison of index-based solutions for reachability query processing.

**Method**	**Index size**	**Construction time**	**Query time**	**Approximate**
Interval labeling	High	High	Low	-
Hop labeling	Medium	High	Medium	-
Online search	Low	Low	High	-
Backbone	Medium	High	Medium	-
ActiveReach	Low-high	Low-high	Low	✓

### 2.2 Graph embedding

Many approaches are proposed to represent graphs in a low dimensional space, such as Locally Linear Embedding (LLE) (Roweis and Saul, 2000), IsoMap (Tenenbaum et al., 2000), and Laplacian Eigenmaps (Belkin and Niyogi, 2001). These approaches use singular value decomposition (SVD) or principal component analysis (PCA) as a dimension reduction method to learn node representation in low dimensional space. More recently, a category of methods has been introduced, inspired by language models [e.g., Skipgram (Mikolov et al., 2013)] to learn node representations for large-scale graphs. Deepwalk (Perozzi et al., 2014) is one of the pioneering works in this category, which uses uniform random walks to transform graph into node sequences and generates node representations by using the skip-gram model (similar to word2vec). LINE (Tang et al., 2015) is another method in this category which defines two functions as first-order proximity and second-order proximity to capture first-hop and second-hop relational information between vertices. Node2vec (Grover and Leskovec, 2016) utilizes random walks in a bias way to learn node embeddings. The authors study BFS and DFS such as random walks to capture different similarity measures between nodes. Another study that extends Deepwalk to capture higher order proximity is HOPE (Ou et al., 2016). HOPE uses Katz (Katz, 1953) and pagerank (Song et al., 2009) as similarity functions to preserve transitivity while learning node representations. A comprehensive survey on graph embedding methods is detailed in this study (Goyal and Ferrara, 2018). Graph neural networks (GNNs) are also very popular these days to realize node status propagation on graphs. In fact, GNN can be defined as a generalization of traditional convolutional neural network (CNN) models for graph-structure data. For example, a widely used GNN model, graph convolutional network (GCN) (Ying et al., 2018), defines the graph convolution operation on graph nodes. In this study, a graph node collects node status information from its connected neighbors and update its own status. GraphSage (Hamilton et al., 2017) is another study which proposed a method to only sample a fixed size of neighbor nodes for graph convolution with much lower complexity. Graph attention network (GAT) (Velicković et al., 2017) also is another recent work which introduced the attention mechanism into GNN. GAT introduces weighted aggregation of neighbor status on more important neighbor nodes. A comprehensive survey on GNN and its variations is detailed in this study (Wu Z. et al., 2021). In general, most of these solutions embed two nodes closely only if they are close and well-connected in the graph. There is another category of GNNs that leverage position information of nodes to learn their embeddings (You et al., 2019). This type of GNNs is efficient for position-aware tasks where the distance of nodes is important (You et al., 2019).

In contrast with all existing study on graph embedding, ActiveReach embeds graph nodes in reachability space (rather than proximity space), where in the embedded space graph nodes reside in the vicinity of reachable nodes (rather than local nodes) in the original graph. We demonstrate this by experimentation.

### 2.3 Index structure learning

Kraska et al. (2018) introduced the idea that traditional index structures can be improved by learning index structures. The main idea is to learn the distribution and structure of the data to obtain a compact index representation. This study demonstrated that learned models have the potential to provide significant benefits over traditional index structures. For example, in Mitzenmacher (2018), it is shown that a Bloom filter can be used as a binary classifier predicting whether a key exists in a set. In Ortiz et al. (2018), authors proposed the idea of training a deep learning model to predict query cardinalities. Instead of using basic traditional statistics about data distribution to estimate cardinalities, in this study a model is trained to learn the main properties of the data to learn sub-query representations used to determine the cardinality of different types of queries for query planning. To the best of our knowledge, our study is the first to introduce a learned index for reachability query processing on graphs.

### 2.4 Active learning

Active learning is a strategic approach that intelligently selects specific data points for labeling to optimize model performance. This method has received extensive attention and study due to its efficacy in enhancing model accuracy (Settles, 2009). In recent years, GNNs have become popular in various graph learning tasks, such as node classification or link prediction, as highlighted in recent studies (Goyal and Ferrara, 2018). However, a common challenge arises from the impracticality of labeling all nodes or edges in many cases, which notably impacts the performance of GNNs. While active learning has been applied for addressing low label ratios in various data types such as text and images, adapting it to work effectively with graph-structured data is challenging (Settles, 2009).

Recently, there has been some research focusing on active learning in graphs. In Gao et al. (2018) and Cai et al. (2017), an active learning strategy is introduced for node classification. A node selection strategy that combines multiple criteria, including graph structural information and model uncertainty, is introduced. Another study (Wu Y. et al., 2021) also proposed different selection criteria and applied a multi-armed bandit method to learn the weights of the selection metrics dynamically. None of the prior research has proposed methods for actively selecting informative pairs of nodes to capture maximum reachability.

## 3 Problem definition

In this section, we define several concepts, including the concept of approximate reachability query.

### 3.1 Exact reachability query

In graph *G*, a reachability query between pair of vertices (*u, v*) returns true to indicate the vertices are reachable if there exists a path (*p*_*u, v*_) from *u* to *v*.

### 3.2 Transitive closure

Let *G* = (*V, E*) be a graph, where *V* and *E* are the set of vertices and edges, respectively. Matrix *A* is the reachability matrix or transitive closure (TC) of *G*, where if node *u* can reach node *v* in *G*, then the corresponding element *A*_*uv*_ = 1, otherwise *A*_*uv*_ = 0.

### 3.3 Approximate reachability query

Given a graph *G*, sparsity ratio α, and a pair of nodes (*u, v*) in *G*, approximate reachability query returns the probability that *v* is reachable from *u*. The sparsity ratio α (0 ≤ α ≤ 1) and the accordingly query budget *b* (*b* = α|*V*|^2^ distinct pairs of nodes) are user-defined parameters that determine the number of pair of nodes to be labeled to enable learning approximate reachability. With *b*, user can limit both the memory size and the amount of time an approximate reachability query processing method can use to partially pre-compute TC toward learning an index structure to answer approximate reachability queries on the entire graph *G*. In this study, |*V*|^2^ is the size of the reachability matrix *R* of the graph *G*. In our implementation, we call the graph generated from the reachability matrix as reachability grap. We use reachability matrix and reachability graph interchangeably in the study.

## 4 Preliminaries

### 4.1 GCN

Given a graph *G* = (*V, E*) with *N* nodes *v*_*i*_∈*V*, edges (*v*_*i*_, *v*_*j*_)∈*E*, an adjacency matrix *A*∈*R*^N*N^, a degree matrix *D*_*ii*_ = ∑*A*_*ij*_, a node feature matrix *X*∈*R*^N*F^, GCN (Kipf and Welling, 2017) is an efficient variant of convolutional neural networks (CNN), operates directly on graphs, using their structural data. It can address the task of node classification within a graph where labels are only provided for only a limited portion of nodes (semi-supervised learning). Specifically, the architecture of aggregation is summarized as


(1) $$H^{\left(l+1\right)}=\sigma ({\tilde{D}}^{-\frac{1}{2}}\tilde{A}{\tilde{D}}^{-\frac{1}{2}}H^{l}W^{l})$$

GCN has made two main improvements: A self-connection is added to each node in the adjacency matrix, and the adjacency matrix is then normalized based on the degrees. We finally obtained Ã and $\tilde{D}$. *H*^*l*^ represents the embedding of the nodes in *l*_*th*_ layer, *W*^*l*^ represents the weight matrix for the *l*_*th*_ layer, and σ represents the non-linearity.

### 4.2 Position-aware embedding

One of the limitations of current GNN architectures is their inability to encode the positional information of nodes within the graph structure. This gap is addressed by Position-aware graph neural networks (P-GNNs). position-aware graph neural networks (P-GNNs) (You et al., 2019) represent a novel category of graph neural networks designed to produce node embeddings by incorporating a node's distance information relative to all other nodes in the graph.

This is how P-GNN generates a node's embedding. P-GNN initially chooses several node sets known as anchor sets. Next, it trains a non-linear aggregation method, which utlizes node feature data from each anchor set and adjusts it based on the distance between the node and the anchor set. A P-GNN consists of multiple P-GNN layers. In particular, in the *l*_*th*_ P-GNN layer, it begins by sampling *k* random anchor sets *S*_*i*_. Every dimension of the embedding is determined through a process involving three steps: initially computing the message from each node in the anchor set using a message computation function *F*, applying a message aggregation function, and ultimately subjecting the result to a non-linear transformation to obtain a scalar, achieved through weights *w* and non-linearity σ (You et al., 2019).

## 5 Baseline solutions

In this section, we outline baseline solutions, including a naive approach and the adoption of existing reachability processing methods to provide an approximate version.

- *Naive solution*: As a naive solution for partial computation of *R* with query budget *b*, one can select *b* pairs of nodes from the graph uniformly at random. BFS is then executed between selected pairs of nodes to compute reachability in the matrix. This algorithm is referred to as random pair sampling (RP).
- *Tree cover*: This family of reachability methods uses online search to answer reachability queries by computing labeling information to efficiently prune the search space (Yildirim et al., 2010; Jin et al., 2008; Veloso et al., 2014). One of the representative methods in this category is GRAIL (Yildirim et al., 2010) which uses min-post labeling directly on the input graph *G*. In the experiments, we propose an approximate solution to select most reachable vertices for partial computation of *R* based on the GRAIL approach. We generate diverse spanning trees which have various reachability information using multiple DFS traversals with limited length *l*. The starting vertex of DFS is selected randomly. The order of traversal of the children changes proportional to their sum of indegree and outdegree. We start DFS traversal within length of *l* until *b* elements of matrix *R* are computed.
- *2-Hop labeling*: As explained in Section 2, hop labeling methods (Yano et al., 2013; Jin and Wang, 2013; Wei et al., 2014) utilize intermediary vertices for recursively encoding reachability. We adopt and adapt this approach for partial computation of *R* by executing multiple depth limited BFS traversals from selected nodes *u* and compute the corresponding element in matrix *R* if reachability to any of its children *v* is not computed yet. If vertex *v* is reachable from a node whose reachability is already computed, *v* is pruned.
- *Landmark*: This family of methods reduces the size of the original graph by extracting recurrent reachability information to form a reachability backbone. We select SCARAB as a representative method from this family (Jin et al., 2012). To introduce an approximate backbone-based method, we extract the reachability backbone based on the concept of vertex cover. The vertex cover of the input graph *G* is a set of vertices *S*, where for each edge (*u, v*)∈*E*, we have ({*u, v*}∩*S*)≠∅ (Cheng et al., 2012). A vertex cover *S* is called a minimum vertex cover if it is the smallest vertex cover among all vertex covers of *G*. To compute *b* elements of the reachability matrix *R*, we first compute a 2-approximate minimum vertex cover *S* of the input graph *G*, and then, we perform BFS on *G* to determine reachability between each pair of vertices *u, v*∈*S* until *b* elements of matrix *R* are computed.
- *Forest fire sampling*: In Forest Fire sampling (Leskovec et al., 2005), we enhance the sampling process by prioritizing vertices with higher degrees, contrasting with the naive approach of random pair sampling. This method begins by randomly selecting a vertex *v* and then probabilistically including some of its outgoing edges to neighboring nodes. The number of selected neighbors is chosen from a geometric distribution with a mean of $\frac{p_{f}}{\left(1-p_{f}\right)}$, where *p*_*f*_ is called the burning probability. We adopt this sampling method as follows. We define the probability for a vertex *i* to be selected to be proportional to its Pagerank score (Song et al., 2009) in the graph and this process continues until the query budget is exhausted.

## 6 ActiveReach

**Figure 1:**
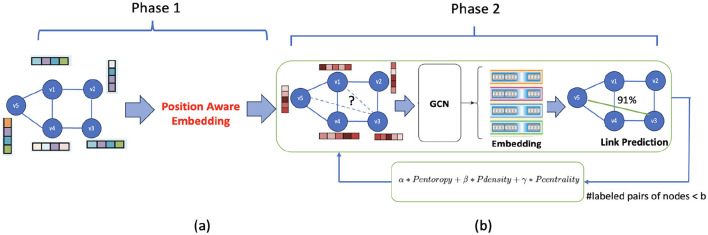
ActiveReach overview: **(a)** Attributed graph as input and generating reachability aware embedding using position-aware embedding. Here, the vectors correspond to the original nodes' attributes. **(b)** Reachability graph utilizes GCN to predict reachability and leveraging active learning to select informative pairs of nodes to generate the partial reachability graph. The vectors assigned to nodes here represent the new attributes generated from the position-aware embedding.

Figure 1 depicts our proposed framework for approximate reachability query processing, dubbed ActiveReach. Transitive closure of *G* can be represented as a reachability graph, where reachability prediction means predicting links between nodes within this graph. Reachavility graph is constructed from transitive closure, where if node *u* can reach node *v* in *G*, then the corresponding element is 1, otherwise 0. The end-to-end solution works as follows. We begin by partially populating the reachability graph which allows us to predict reachability for the entire graph. To compute the reachability graph partially, given a query budget one can randomly select pairs of nodes from the original graph to calculate reachability (baseline solution). After computing partial reachability graph, we can then use graph embedding techniques to learn the embedding of the nodes in the partial reachability graph, which we call “reachability embedding.” Finally, we can use link prediction methods to predict reachability for the remaining portion of the graph.

However, the baseline solution described above has two issues. First, the reachability graph only provides connectivity data, lacking sufficient information for reachability learning. In particular, the reachability graph does not have information about node attributes in the original graph, not about placement of the nodes in the original graph, both of which are important for accurate reachability estimation.

For example, in social networks, as demonstrated in Grover and Leskovec (2016), nodes with similar attributes show a higher probability of reachability due to *homophily*. Moreover, there is likely a decrease in the probability of reachability as the distance between nodes increases in the original graph. To this end, in Phase 1 of our proposed solution, we enhance the reachability graph by incorporating nodes' attributes and positional information as shown in Figure 1a. Second, random selection of the pairs of nodes to generate the partial reachability graph is not an effective sampling approach to inform the link prediction methods that predict the reachability during the query time. Instead, in the second phase of ActiveReach, we propose an active learning solution to intelligently select the most informative pairs of nodes to capture maximum amount of reachability information from the selected pairs to best inform prediction of the links in the reachability graph during query time, as illustrated in Figure 1b.

To further elaborate, with ActiveReach, we learn reachability indexes in two phases. First, given an attributed graph *G*, we apply P-GNN to learn node embedding utilizing node's attributes and positional information in *G* as shown in Figure 1a. The generated embedding is used as the node's attributes in the reachability graph. Second, starting from a reachability graph with new node's attributes and small set of initial labeled pair of nodes, we apply a graph embedding (GCN) to learn reachability. In each iteration, we leverage active learning to select informative pair of nodes to calculate reachability if the query budget is not reached as shown in Figure 1b. With ActiveReach not only we can learn an index to predict approximate reachability between pairs of query nodes but also generate a reachability embedding space where each node in *G* is represented by a vector in the embedded space. The reachability embedding space can be used for other downstream learning tasks beyond approximate reachability query processing. In general, we collect our training set (based on the query budget) in the active learning manner and learn reachability. During online query processing, we return the probability of reachability for a given pair of nodes. Next, we explain each phase in detail.

### 6.1 Reachability aware attributes

The reachability graph, derived from the reachability matrix, lacks sufficient information on its own. Reachability graph is not aware of nodes' attributes and positional data. Since reachability computation requires positional awareness, employing structure-aware GNNs proves inefficient for this task. To improve this, we utilize P-GNN to enrich nodes in the reachability graph with new attributes preserving their positional information in the original attributed graph *G*. In the first phase, P-GNN combines both node attributes and positional data to generate reachability-aware embedding for each node to enhance reachability learning in the second phase.

### 6.2 Active learning strategy

To compute reachability matrix given query budget *b*, we need to select *b* pairs of nodes to calculate reachability and generate a training data set. Since computing reachability is time and memory expensive, we propose an active learning strategy to intelligently choose the pairs of nodes to label them to capture maximum reachability information from the graph with a fixed query budget *b* shown in Figure 1b. The more informative pair of nodes selected, the higher the accuracy of the generated reachability index in approximate reachability query answering. To compute reachability and label pair of nodes, we use BFS as simplicity. Any reachability computation method suitable for a given graph structure can be used in this step.

#### 6.2.1 Model prediction uncertainty

In this study, we explore uncertainty, a widely adopted technique in active learning literature. By employing uncertainty metrics, we can select pairs of nodes where our classification model shows the highest uncertainty. Entropy calculates a measure of uncertainty in predicting reachability for node pairs *v*_*i*_ and *v*_*j*_. Information entropy is calculated as follows:


(2) $$entropy(v_{i},v_{j})=\sum_{c=1}^{C}P(Y_{ij_{c}}=1)log(P(Y_{ijc}=1))$$

where *P*(*Y*_*i*_*j*__*c*__ = 1) is the probability of path *v*_*i*_, *v*_*j*_ belonging to class *c* predicted by path prediction. The larger value for entropy indicates our model is more uncertain about reachability of *v*_*j*_ from *v*_*i*_.

#### 6.2.2 Embedded space information

One challenge to select the most informative pairs of nodes only based on the model uncertainty is that we might find noises and outliers which are not representative. Using uncertainty metrics may lead us to explore unrepresentative regions of the graph. Here, we introduce a selection measurement based on the reachability embedding space. This parameter selects pairs of nodes which are most representative in the embedded space. Information density is higher in the dense regions of embedded space. Computing reachability between nodes from each dense region has reachability information from parts of graphs with different reachability patterns. To this end, we first apply K-means on the embeddings of all unlabeled nodes and second calculate the Euclidean distance between each node of the cluster and the cluster center. The pairs of nodes closer to the center of their clusters are selected. Here, we show how we calculate distance of node *v*_*i*_ to the center of embedding cluster (CC).


(3) $$\begin{array}{l} distance\left(v_{i}\right)=\frac{1}{1+Euc\left(embed_{v_{i}},CC_{v_{i}}\right)} \end{array}$$

(4) $$\begin{array}{l} density\left(v_{i},v_{j}\right)=distance\left(v_{i}\right)+distance\left(v_{j}\right) \end{array}$$

where Euc() is the Euclidean distance, *embed*_*v*_*i*__ is the embedding of node *v*_*i*_ and *CC*_*v*_*i*__ is the center of the cluster that *v*_*i*_ belongs to. After the computation of node distances to their respective clusters in the embedded space, we select pairs of nodes *v*_*i*_ and *v*_*j*_ characterized by the highest distance sum.

#### 6.2.3 Graph structure

To select nodes which are most informative in terms of reachability in graph, we select the pairs of nodes based on their importance based on the graph structure. For instance, reachability between nodes locate on multiple shortest paths is very critical. The graphical structure is then calculated to measure representativeness for unlabeled pair of nodes. There are various metrics in the literature (Song et al., 2009) measuring the importance of nodes (e.g., degree, PageRank, closeness, and betweenness centrality). In this study, we use betweenness of nodes to show how they are important in the graph. Betweenness centrality (Kintali, 2008) of a node *v*_*i*_ is the number of the shortest paths that *v*_*i*_ is part of.


(5) $$\begin{array}{l} betweenness\left(v_{i}\right)=\frac{\sigma_{v_{i}}}{\sigma } \end{array}$$

(6) $$\begin{array}{l} centrality\left(v_{i},v_{j}\right)=betweenness\left(v_{i}\right)+betweenness\left(v_{j}\right) \end{array}$$

where σ_*v*_*i*__ is the number of shortest paths *v*_*i*_ belongs to, and σ is all the shortest paths. After computing betweenness, we select pairs of nodes *v*_*i*_ and *v*_*j*_ characterized by the highest betweenness sum.

#### 6.2.4 Combination of different criteria

To have a fair comparison between different selection criteria and make scores comparable, we need to normalize scores. To this end, we convert them into percentiles as in Zhang et al. (2016). Denote Percentile (u,v) as the percentile of pair of nodes in terms of each metric. We define the selection criteria to select the pair of nodes for labeling as follows:


(7) $$\begin{array}{l} \alpha *P_{entoropy}+\beta *P_{density}+\gamma *P_{centrality} \end{array}$$

where α+β+γ = 1.

#### 6.2.5 Complexity analysis

Using position-aware embedding in phase 1, typically each anchor set contains m nodes, therefore there are *O*(*mnlog*^2^*n*) message communications because every node communicates with *O*(*log*^2^*n*) anchor sets in a graph with n nodes and e edges. Based on the You et al. (2019), for each anchor set, we only aggregate message from the node closest to a given node *v* which eliminates the factor m in the complexity of position-aware embedding, reducing the complexity to *O*(*nlog*^2^*n*) (You et al., 2019). In phase 2, the number of communications is *O*(*ne*) for the graph embedding. K-means time complexity is *O*(*n*^2^). The overall complexity is *O*(*ne*).

## 7 Experimental evaluation

In this section, we will first present our experimental methodology, and then, we will review our experimental results focusing on reachability prediction performance, ablation study, and parameter sensitivity analysis for the proposed solution.

### 7.1 Datasets

We performed our experiments using both real and synthetic datasets. Specifications of the selected real datasets are illustrated in Table 2.

- *Cora* This dataset is a citation graph (Sen et al., 2008). Each document is represented as a node, and if one document cites another, there is a citation between them. A bag-of-words embedding for each document is used as a feature set.
- *Pubmed* This dataset is also a citation graph (Sen et al., 2008), where each citation link between documents is represented as an edge. Each document in the graph is equipped with a bag-of-words embedding, serving as its node features.
- *Flicker* In the Flickr dataset (Zeng et al., 2020), each node corresponds to a user, while an edge denotes a follow relationship between two users. Node features are derived from the 500 most common tags associated with their photos.
- *Yelp* dataset denotes an active user on Yelp (Zeng et al., 2020), where edges between nodes indicate friendship relationships. Node features have details from user reviews. Utilizing word2vec (Mikolov et al., 2013), the reviews are transformed into 300-dimensional vectors and used as node features.
- *Reddit* Reddit^1^ In this graph, nodes represent posts from users, and an edge is assigned between two nodes if the corresponding users have posts on the same topic. This data is from Reddit posts in September 2014. The embedding of the post title, the score of the posts, and the number of comments on each post are combined and used as features for each node.
- *DBpedia* For DBpedia,^2^ each vertex represents an entity, and each edge denotes a relationship between two entities. The keywords of each entity are embedded and used as features.

**Table 2:** Statistics on real datasets.

**Datasets**	**Nodes**	**Edges**	**Avg degree**	**Features**
Cora	19,793	126,842	6.4	1,433
PubMed	19,717	88,648	4.4	500
Flicker	89,250	899,756	10	500
Yelp	716,847	13,954,819	19.4	300
Reddit	232,965	114,618,780	492	602
DBpedia	8,099,955	71,527,515	8.8	15

### 7.2 Query budget *b*

We choose the query budget *b* between 5% to 30% of |*V*^2^| for a given dataset. With smaller values of *b*, the performance of the algorithms will be lower.

### 7.3 Alternative solutions

We evaluate performance of ActiveReach comparing with representative index-based solutions from the related study (Zhang et al., 2023). We discussed the adapted version of traditional index-based solutions in Section 5. We use index-based solutions to complete reachability matrix given a query budget *b*. We then apply GCN (Ying et al., 2018) on the reachabity graph to generate embeddings. Here, we explain the parameters we used for baseline solutions.

- *Tree cover*: We use fast online search (GRAIL) with random selection of starting nodes and DFS length *l* = 40.
- *2-Hop labeling*: We use 2-hop labeling with random selection of starting nodes and BFS length of *l* = 20.

### 7.4 Reachability prediction performance

To evaluate the performance of ActiveReach, first we measure reachability prediction performance. For GCN, we follow the experimental setup used in state-of-the-art semi-supervised graph embedding methods (Ying et al., 2018). The number of initially labeled pairs of nodes is set as 40, as used in Ying et al. (2018). We used the setups from You et al. (2019) to implement position-aware embedding for the attributed graphs. In particular, after generating embeddings, we learn a classifier that can receive the embedded vectors of two nodes and predict the existence of a path between them in the original graph. The probability of reachability is generated by our classifier. To achieve this, after learning the reachability embedding of vertices, given two nodes *u* and *v*, we predict the existence of a path between them (*path*_*u, v*_) by learning a classifier *f*(*x*), where *x* is the set of features for *path*_*u, v*_. We use Hadamard (Grover and Leskovec, 2016) as a binary operator for concatenating the embedding vectors. Random Forest with 100 trees and a maximum depth of 10 is used as the classifier. We consider threshold of 0.5 for our binary classifier. BFS is used to compute reachability for the pair of nodes selected from the active learning strategy. Note that each method is executed 100 times, and average result is reported. In this experiment, we show how increasing the query budget impacts reachability accuracy.

**Figure 2:**
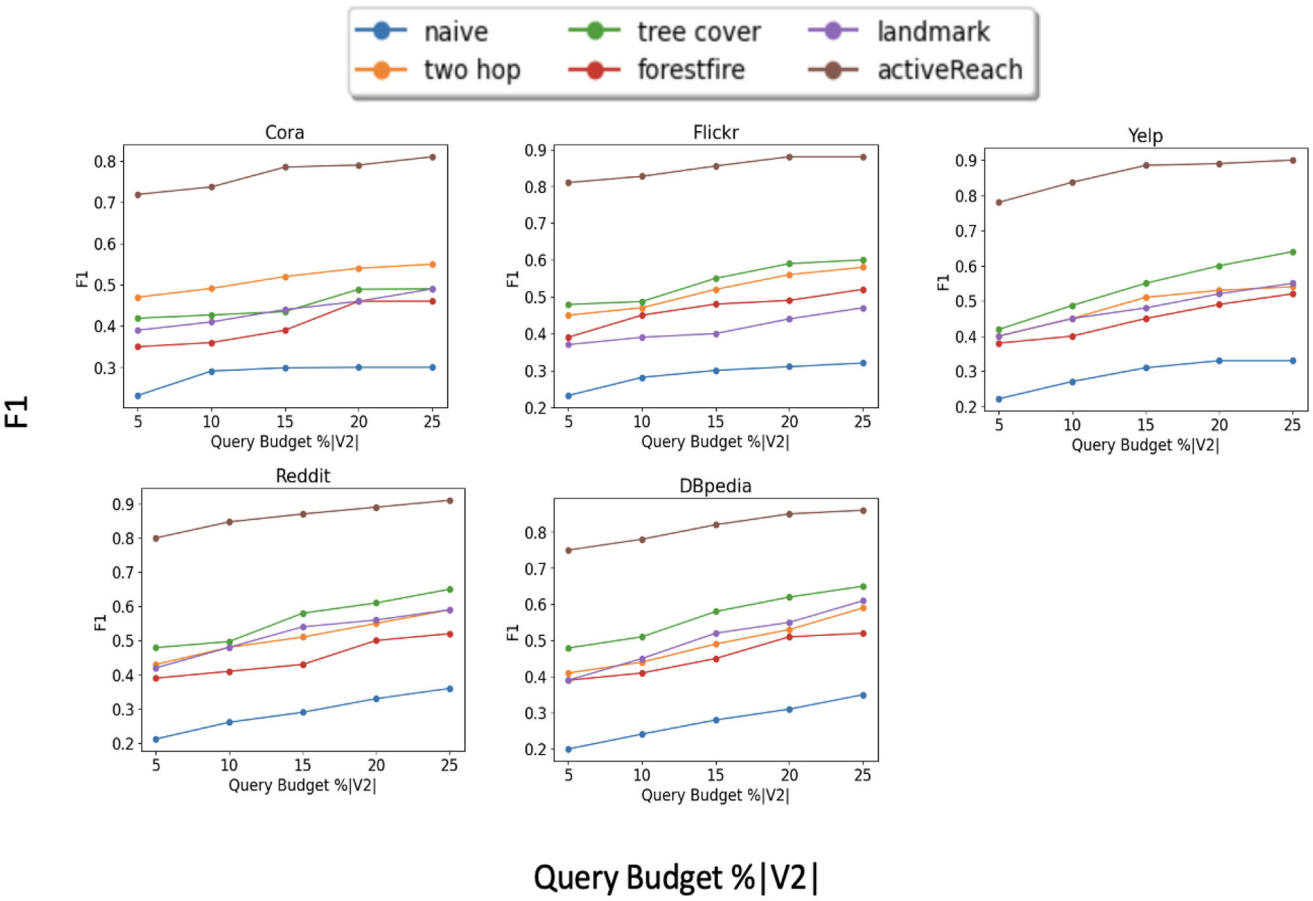
Reachability prediction performance (F1).

Figure 2 illustrates the results for each dataset. We do not show results for the Pubmed dataset because its results are similar to those of the Cora dataset. From the figure, ActiveReach shows the best performance across all datasets, especially for the Cora dataset, which has a very large number of attributes per node. For the Reddit dataset which has a very high average degree (dense dataset), ActiveReach performs very well. This is because in dense datasets, most nodes have access to distant nodes due to the high average degree, increasing the chance to learn reachability information from neighbors. From the Figure, we also observe that tree cover (GRAIL) has the best performance among index-based and sampling solutions and it does so with a small DFS length. GRAIL does not work well for the Cora data set which is sparse. Overall, 2-hop labeling does not perform well because it uses its query budget mostly traversing a local neighborhood especially for dense datasets. In addition, Baseline, Landmark, and ForestFire are among the worst performing methods to capture reachability information with all datasets. Landmark only computes reachability between minimum vertex covers and graph embedding cannot predict the reachability of pairs of nodes with limited neighborhood information. In addition, Forest Fire is not able to capture enough reachability information from nodes located far apart in the input graph, especially in sparse graphs. Overall, ActiveReach which utilizes nodes' attributes, position-aware embedding, and active learning strategy performs better. ActiveReach uses its budget to cover representative nodes from various parts of the graph while capturing enough local reachability information. This demonstrates that both local and global graph structure as well as nodes' attributes are essential to characterize the graph reachability, as expected.

**Figure 3:**
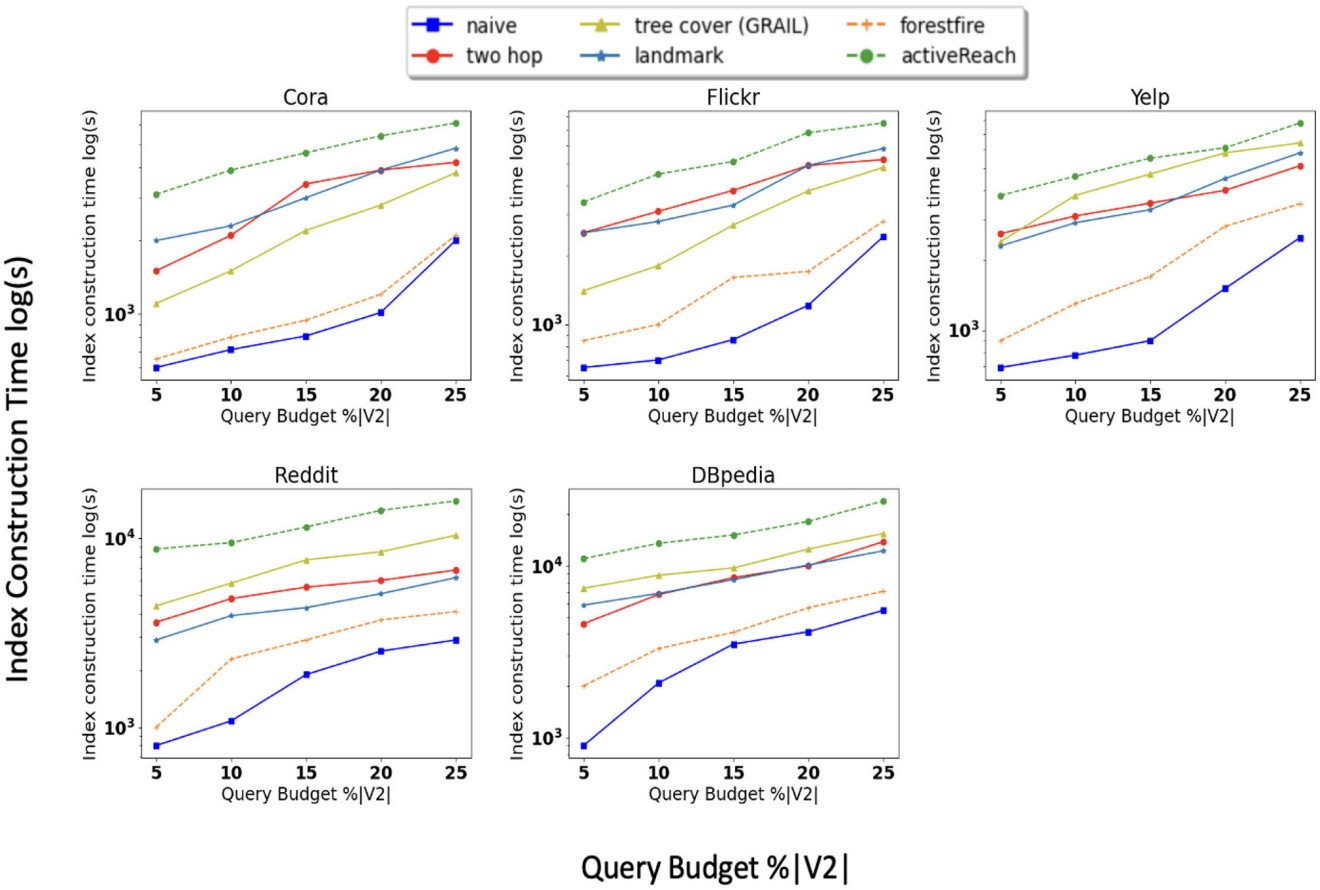
Index construction time.

In addition, we compare the time it takes for each method to partially compute matrix *R*. Figure 3 shows that ActiveReach takes more time due to the position-aware embedding and active learning strategy. From index-based solutions, tree cover (GRAIL) takes longer to compute *R* especially in dense graphs. Landmark and Forest Fire spend longer to compute the matrix for sparse datasets in comparison with dense datasets. It is worth noting that the query time, or inference time, is minimal and remains unaffected by the query budget.

**Figure 4:**
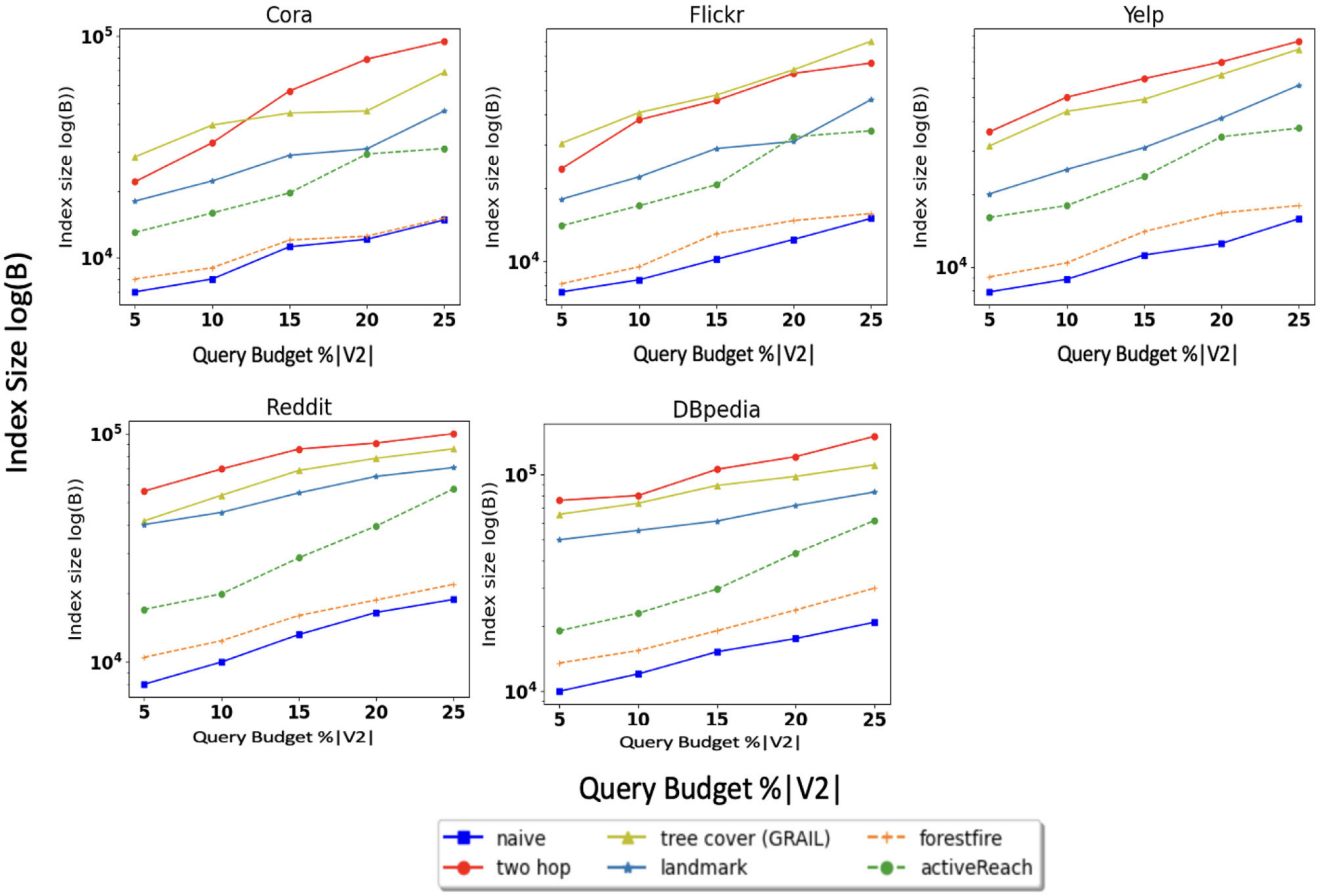
Index size.

We also evaluate memory consumption during execution of these methods; the results of this experiment are shown in Figure 4. Tree cover (GRAIL), 2-hop labeling, and Landmark have larger memory footprints compared to other methods. For instance, creating multiple tree-cover in GRAIL and minimum vertex-cover in Landmark, which are both time and memory consuming, are at the core of these algorithms.

### 7.5 Active learning performance

**Figure 5:**
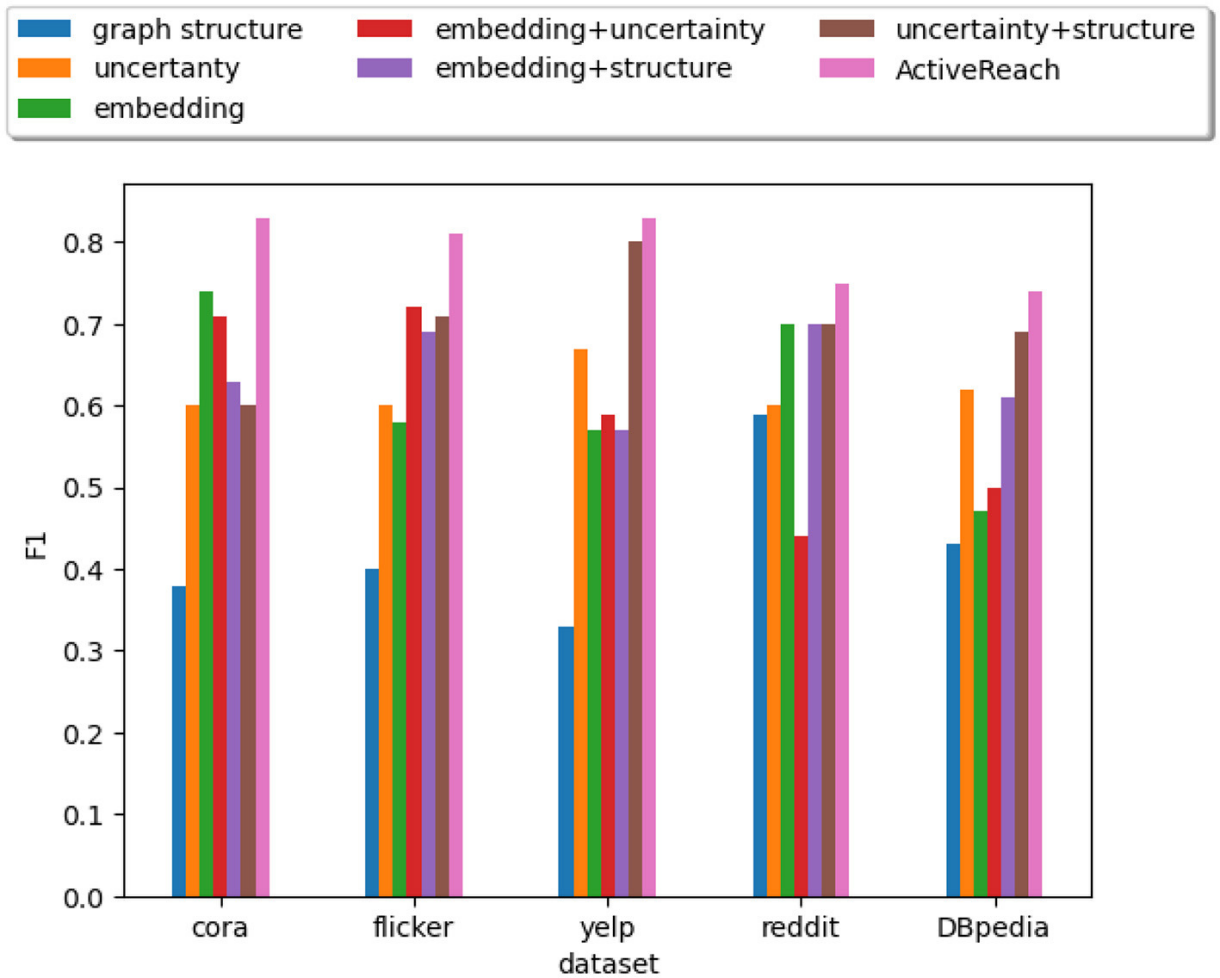
Different active learning strategies.

As mentioned before, ActiveReach intelligently selects representative pairs of nodes to complete the reachability matrix. We consider different combinations of active learning strategies as shown in Figure 5 while fixing the query budget at 0.05%|*V*^2^|. As illustrated in the figure, we cannot preserve enough reachability information with only using graph structural information. Structural information works well only for dense graphs such as Reddit and DBpedia datasets. With utilizing embedded information especially for sparse graphs (Cora), better results are achieved. The reason is that in sparse graphs such as Cora, nodes' attributes and position information from nodes apart from each other are very important to learn reachability which are captured from the embedding strategy. In datasets with a small number of attributes such as DBpedia, the embedding strategy has less impact in comparison with the structural strategy. In addition, it is worth mentioning that depending on the graph structure, active learning strategies can vary significantly. For instance, the choice of clustering method is crucial in sparse graphs, while computing betweenness centrality can be challenging in dense graphs. ActiveReach offers flexibility for users to choose the optimal query budget and active learning strategy according to the specific application and characteristics of the input data.

**Figure 6:**
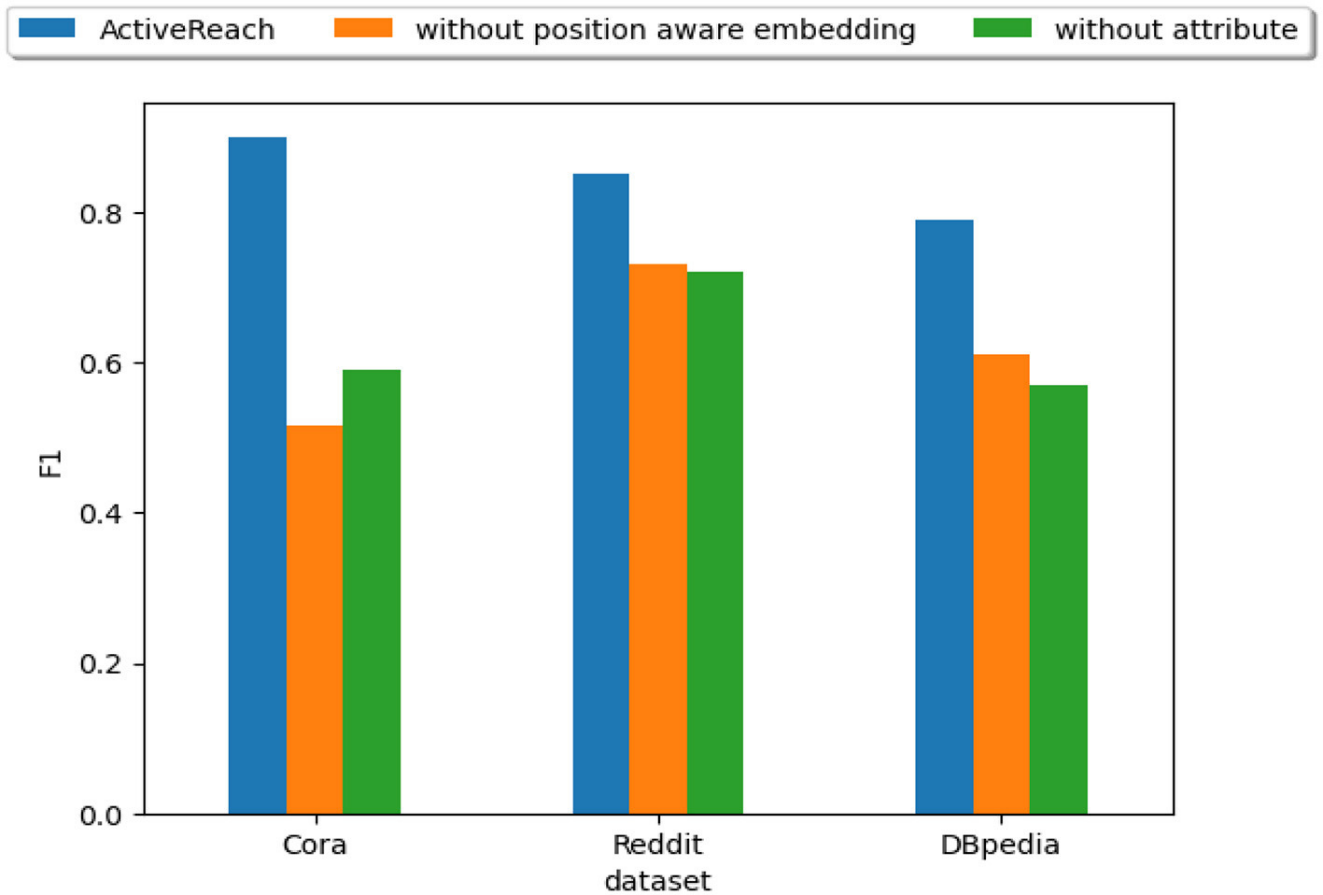
Position-aware embedding and attributes.

In Figure 6, we show the impact of node's attributes and position-aware embedding on the reachability prediction performance.

The figure illustrates that our method does not work well without nodes' attributes especially for sparse graphs such as Cora. Nodes' attributes preserve similarity for distant nodes which is hard to capture their reachability information in sparse graphs. However, the impact of nodes' attributes is less for dense graphs such as Reddit. In addition, position-aware embedding preserves distance of nodes and this information is essential for graphs with large diameter such as DBpedia. Leveraging position-aware embeddings works very well for community graphs such as Reddit which reachable nodes are closer to each other. Adding attributes works best for citation networks that encode keyword embeddings as a similarity measure to preserve reachability.

### 7.6 Parameter sensitivity study

With our next experiment, we evaluated the impact of the data parameters and method parameters. For this experiment, we use the synthetic dataset explained in Table 3 to have more control on the graph structure.

**Table 3:** Statistics on synthetic datasets.

**Datasets**	**Nodes**	**Edges**	**Avg Dag**
rand10m	10M	20M	2
rand10m	10M	50M	5
rand10m	10M	100M	10

**Figure 7:**
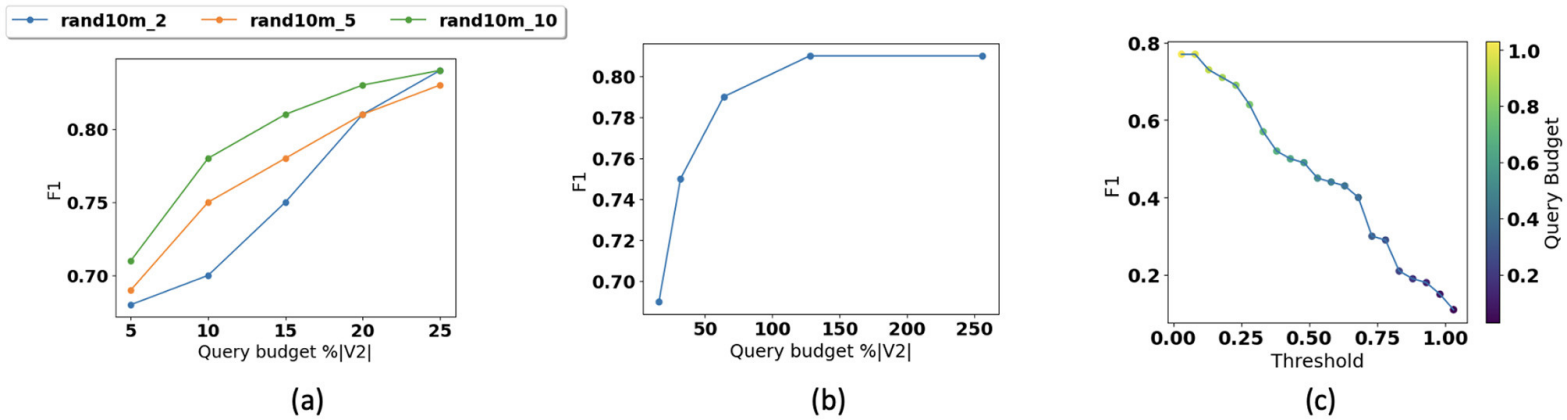
Parameter sensitivity: **(a)** accuracy vs. graph density, **(b)** accuracy vs. dimension, and **(c)** accuracy vs. threshold.

Figure 7 illustrates how changing (Figure 7a) graph density and (Figure 7b) number of dimensions of the embedding space affects the accuracy. We fix the query budget at 0.05%|*V*^2^| and use RandomForest with 100 trees and a max depth of 10. In Figure 7a, we show how performance changes for datasets with different densities. As most of the query budget is used for local traversal in dense datasets, with smaller query budgets path prediction accuracy is higher in comparison with sparse datasets. We also observe that with larger query budgets, accuracy is higher for sparse dataset. In Figure 7b, we also observe that performance tends to saturate once the number of dimensions of the embedded space reaches approximately 128. In Figure 7c, we also show that how changing the prediction threshold or confidence and query budget impact the accuracy. This figure shows that by increasing the threshold and decreasing the query budget, F1 decreases. Prediction threshold or confidence can be defined as a user-defined parameter. Obviously as the user-defined threshold increases, the performance bar is higher for our solution and the F1 score will decrease. We also show that the notion of the query budget is orthogonal to the concept of prediction confidence. While the prediction confidence is generated at the inference time (online), the query budget determines the time the user would like to invest in construction of the index (off line). Of course, the larger the query budget and the investment, the higher prediction confidence is expected. The threshold can be adjusted depending on the application's sensitivity to reachability predictions. For example, in applications such as influence maximization, lower confidence may be acceptable, while virus propagation detection requires higher confidence levels.

**Figure 8:**
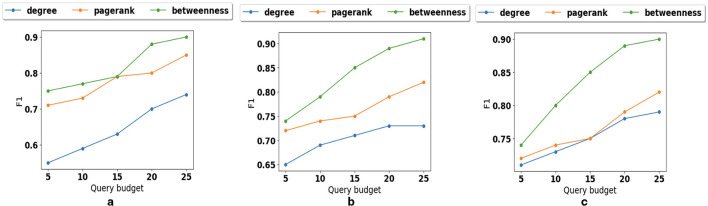
Parameter sensitivity: **(a)** Impact of graph structure for sparse graph. **(b)** Impact of graph structure for medium dense graph. **(c)** Impact of graph structure for very dense graph.

We also examine how different graph structures affect the performance of path prediction. As illustrated in Figure 8a, in sparse graphs, pagerank and betweenness preserve reachability better than degree. The reason is that with small average degree, the neighbor reachability information is not enough. As the density increases (Figure 8b) the impact of node betweenness is higher than pagerank. However, in very dense graphs as illustrated in Figure 8c, node degree shows the importance of nodes and can preserve reachability information similar to pagerank.

## 8 Conclusion and future work

In this study, we introduced ActiveReach, an approximate reachability query processing method. With our proposed method, we learn an index structure to answer approximate reachability queries by partially precomputing reachability information represented by the sparse transitive closure of the input graph. ActiveReach leverages position-aware embedding and nodes' attributes to preserve reachability and intelligently selects pair of nodes to label during training. Our experimental results demonstrate the efficacy and efficiency of ActiveReach in answering approximate reachability queries in real large graphs with limited resources (time and memory). In the future, we plan to extend our proposed framework to process various types of reachability queries including reachability search and top-k reachability.

## Data Availability

The original contributions presented in the study are included in the article/supplementary material, further inquiries can be directed to the corresponding author.

## Footnotes

^1^Available at: http://snap.stanford.edu/graphsage/ (accessed December 2017).

^2^Available at: https://www.dbpedia.org/ (accessed December 2016).
